# Supplementary material for: Organ-specific, multimodal, wireless optoelectronics for high-throughput phenotyping of peripheral neural pathways
Source: Nat Commun. 2021 Jan 8;12:157. doi: 10.1038/s41467-020-20421-8 (PMC7794361; doi:10.1038/s41467-020-20421-8)
Supplement: Supplementary file 5 — Supplementary Information [file 41467_2020_20421_MOESM5_ESM.pdf]

Supplementary Information

Supplementary Figure 1. Device layout and a table of components used for the wireless gastric optogenetic implant.

Supplementary Figure 2. Three-dimensional modeling of the mechanics.

Supplementary Figure 3. Measurement method and equation for various curvatures of a tether.

Supplementary Figure 4. Image of a device and results of cycling test

Supplementary Figure 5. Measurements of optical output power from devices when immersed in saline solution at various temperatures.

Supplementary Figure 6. Measurements of temperature changes as a function of operation duty cycles in wet and dry condition.

Supplementary Figure 7. An experimental assay with computed SAR distributions on a mouse mesh body.

Supplementary Figure 8. Simulation results of electromagnetic couplings induced in an adjacent cage.

Supplementary Figure 9. Detailed information of the proposed 1 × 8 multiple cage wireless power transmission system.

Supplementary Figure 10. Illustration of antenna layouts and distributions of magnetic fields in a cage. Reconstruction of traces of an operating indicator LED.

Supplementary Figure 11. Detailed information of wireless power transfer operation in the cage.

Supplementary Figure 12. Plots of a residual dependence of transmitted power on relative orientation angle between the transmission antenna and the implantable device.

Supplementary Figure 13. Comparison of wireless coverage for the proposed antenna coil and other antenna coil systems.

Supplementary Figure 14. Circuit diagram of the proposed, scalable multimodal wireless gastric optogenetic implant for multiple organ control.

Supplementary Figure 15. Light intensity measurements during varying RF wireless powering of the gastric optogenetic device (n = 5) and varying distances from the LED.

Supplementary Figure 16. Abdominal activation of Calca+ vagal afferent fibers.

Supplementary Figure 17. Schematic illustration of an experimental assay and distributions of electromagnetic field in the assay.

Supplementary Figure 18. Locomotor activity comparison in the assays for open-field and RTPP.

Supplementary Table 1. Summary of procedures for fabrications

Supplementary Table 2. Summary of customized TX antenna specification

Supplementary Figure 1.

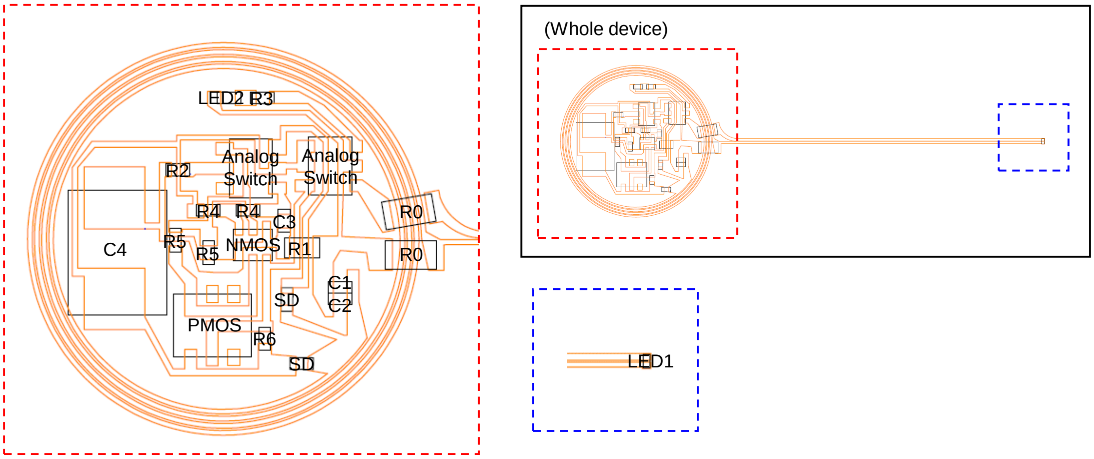

	Components	Product number	Vendor information
**LED1**	460nm, 220 µm x 270 µm x 50 µm	C460TR2227	Cree
**LED2**	632nm, 0.65 mm x 0.35 mm x 0.2 mm	APG0603SEC-E-TT	Kingbright
**SD**	Schottky Diode, 0.65 mm x 0.35 mm x 0.3 mm	DSR01S30SL	Toshiba
**R0**	0 ohm, 1.6 mm x 0.9 mm x 0.55 mm	RCWPM-0603	VISHAY
**R1**	0 ohm, 1.00 mm x 0.55 mm x 0.35 mm	RCWPM-0402	VISHAY
**R2**	0 ohm, 0.65 mm x 0.35 mm x 0.25 mm	RCWPM-0201	VISHAY
**R3**	499 ohm, 0.6 mm x 0.3 mm x 0.23 mm	RC0603F4990CS	Samsung Electro-Mechanics
**R4**	249 kohm, 0.6 mm x 0.3 mm x 0.23 mm	RC0603F2493CS	Samsung Electro-Mechanics
**R5**	10 kohm, 0.6 mm x 0.3 mm x 0.23 mm	RC0603J103CS	Samsung Electro-Mechanics
**R6**	20 kohm, 0.6 mm x 0.3 mm x 0.23 mm	RC0603F203CS	Samsung Electro-Mechanics
**C1**	82 pF, 0.6 mm x 0.3 mm x 0.33 mm	CL03C820JA3NNNC	Samsung Electro-Mechanics
**C2**	330 pF, 0.6 mm x 0.3 mm x 0.33 mm	CL03B331KA3NNNC	Samsung Electro-Mechanics
**C3**	1 µF, 0.6 mm x 0.3 mm x 0.33 mm	CL03A105KP3NSNC	Samsung Electro-Mechanics
**C4**	11 mF, 3.2 mm x 2.5 mm x 0.9 mm	CPH3225A	Seiko Instruments
**NMOS**	1.0 mm x 1.0 mm x 0.34 mm	NTUD3170NZ	ON Semiconductor
**PMOS**	1.7 mm x 1.7 mm x 0.6 mm	NX3008PBKV	Nexperia
**Analog Switch**	1.6 mm x 1.2 mm x 0.5 mm	SN74LVC1G3157	Texas Instruments

Supplementary Figure 1. Device layout (top) and a table of components used for the wireless gastric optogenetic implant (bottom).

Supplementary Figure 2.

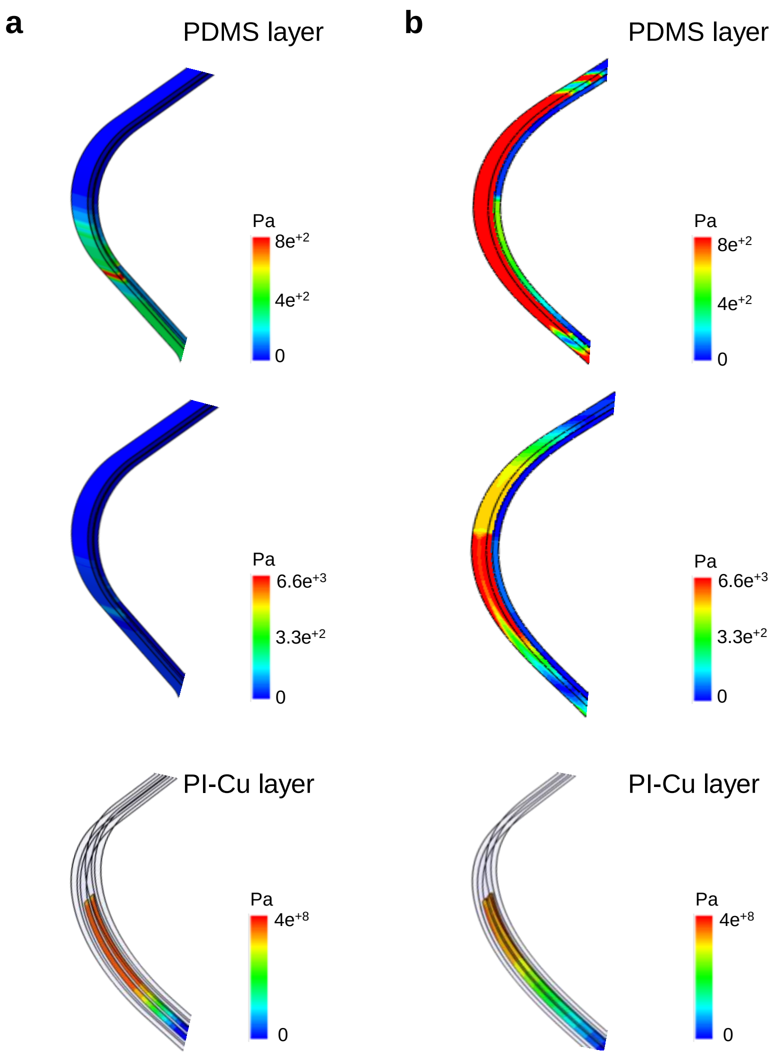

Supplementary Figure 2. Three-dimensional modeling of the mechanics for the pre-curved (**a**) and post-curved (**b**) structure.

Supplementary Figure 3.

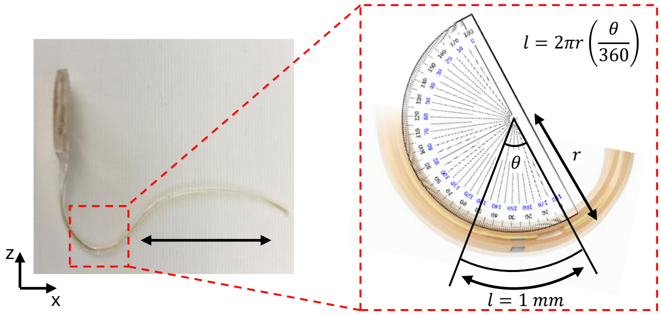

Supplementary Figure 3. Measurement method and equation for various curvatures of a tether.

Supplementary Figure 4.

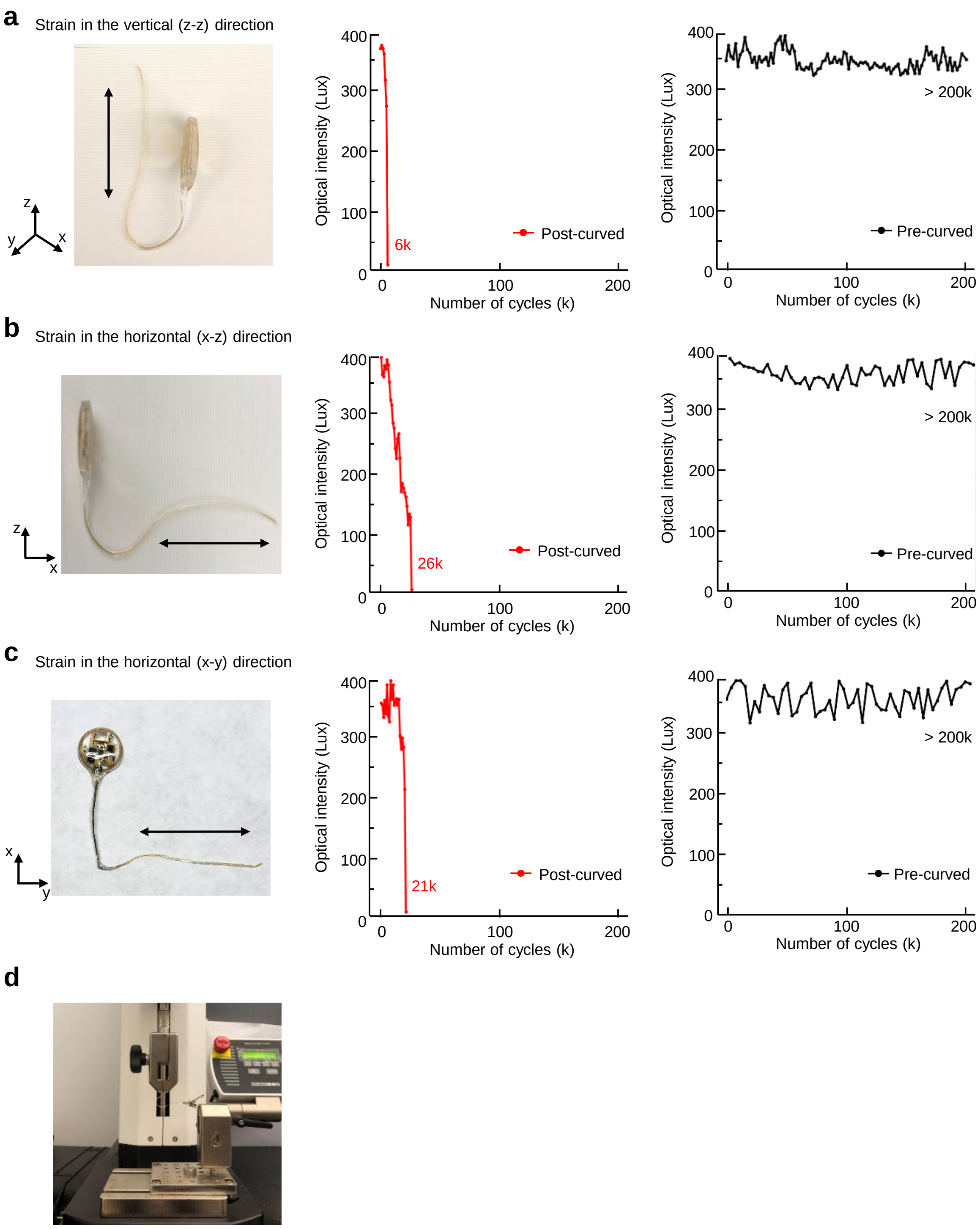

Supplementary Figure 4. Image of a device (left) and results of cycling test for the post-curved (middle) & pre-curved structure (right) when strains applied in the z (**a**), x (**b**), and y (**c**) direction, respectively. Here, an arrow indicate the direction of strains applied to the device. (**d**) Measurement setup for durability test.

Supplementary Figure 5.

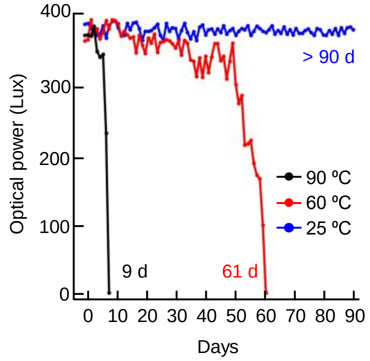

Supplementary Figure 5. Measurements of optical output power from devices when immersed in saline solution at various temperatures, 25 °C, 60 °C & 90 °C.

Supplementary Figure 6.

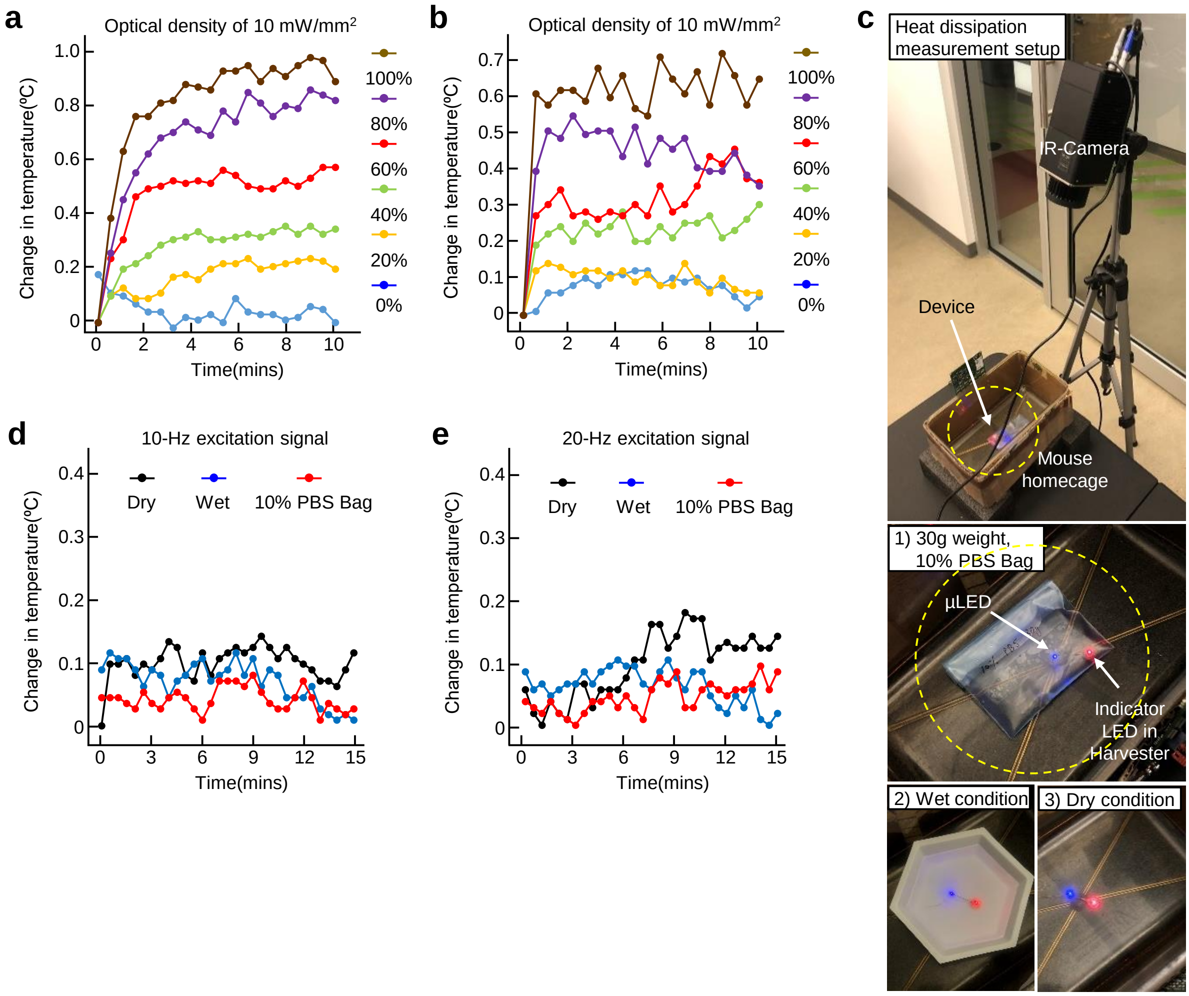

Supplementary Figure 6. Measurements of temperature changes as a function of operating duty cycles in wet (**a**) and dry condition (**b**) using DC power inputs for fixing the optical density of 10 mW/mm^2. Pictures of an experimental setup for wireless measurements of heat dissipation using IR camera (**c**;top). Here, TX power is set to 4 W. The three bottom images show a device mounted on sealed bag of saline solution (10 % PBS), immersed in saline solution, and itself in a cage, respectively. Plots of optical intensity as a function of time at duty cycles (**d**;5 %) and (**e**;10 %) in three different conditions; wet, dry, and PBS bag, respectively. The following provides a guideline for comparison of parameters in experimental settings with those in simulation settings. Magnetic field intensity of 20 A/m in simulation results corresponds to electrical power of 4 W or optical intensity of 10 W/mm^2 in experimental settings.

Supplementary Figure 7.

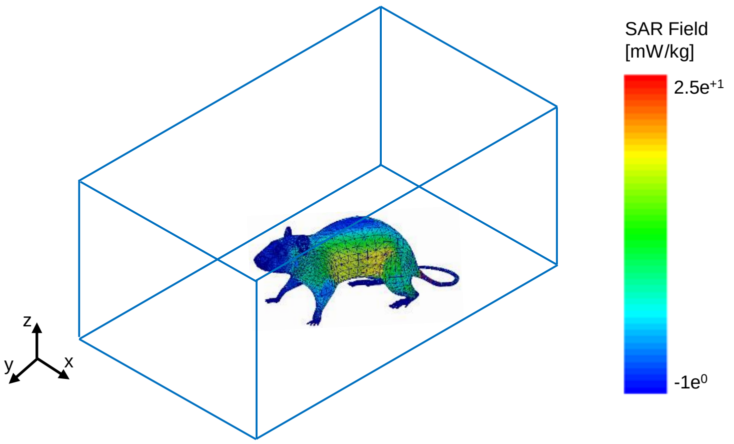

Supplementary Figure 7. An experimental assay with computed SAR distributions on a mouse mesh body.

Supplementary Figure 8.

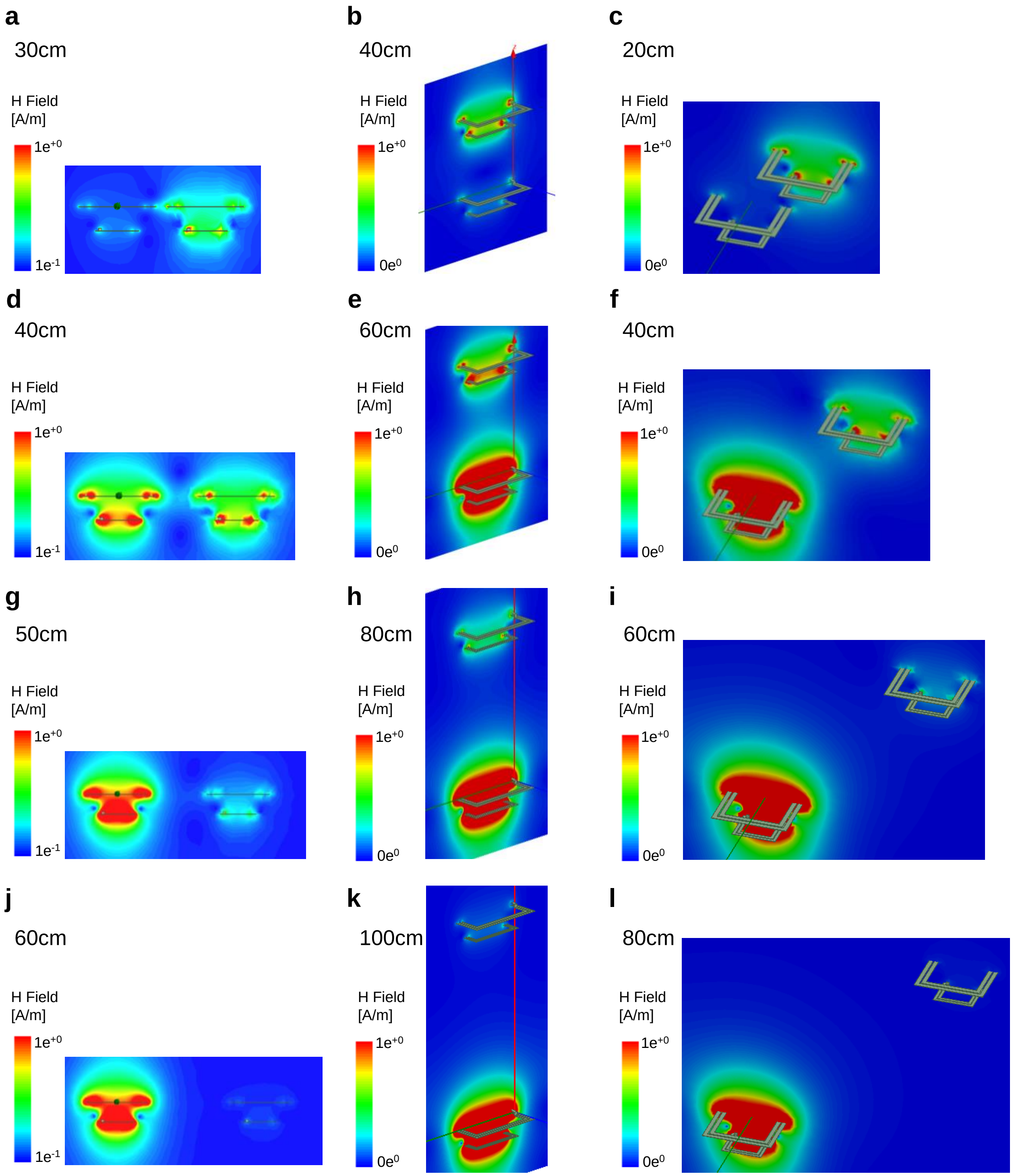

Supplementary Figure 8. Simulation results of electromagnetic couplings induced in an adjacent cage as a function of distance between two cages along the horizontal (**a-d**), vertical (**e-g**), and diagonal (**i-l**) direction, respectively at a transmitted power level of 4 W.

Supplementary Figure 9.

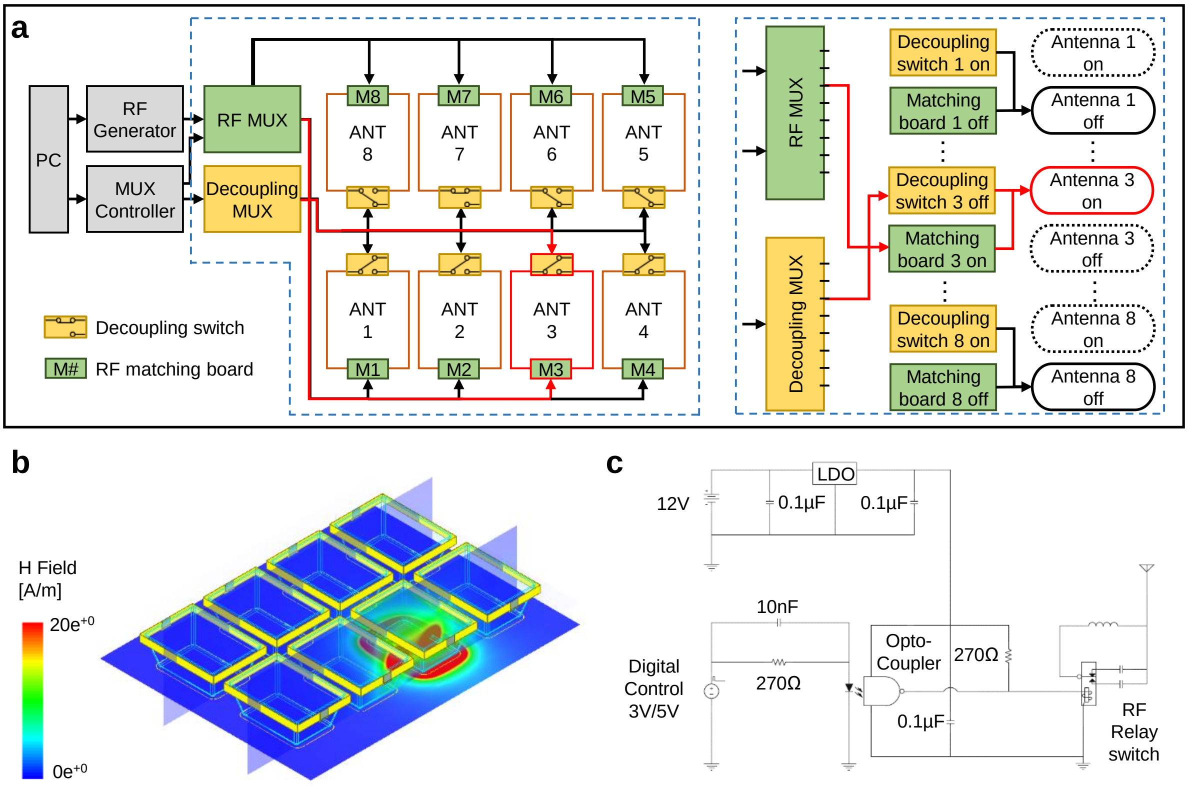

Supplementary Figure 9. (**a**) Functional block diagram of the proposed 1 × 8 multiple cage wireless power transmission system. (**b**) Distributions of electromagnetic field on the top antenna coil, the bottom antenna coil, and vertical direction in each row at a transmitted power level of 4W. (c) Circuit diagram of a unit in decoupling MUX.

Supplementary Figure 10.

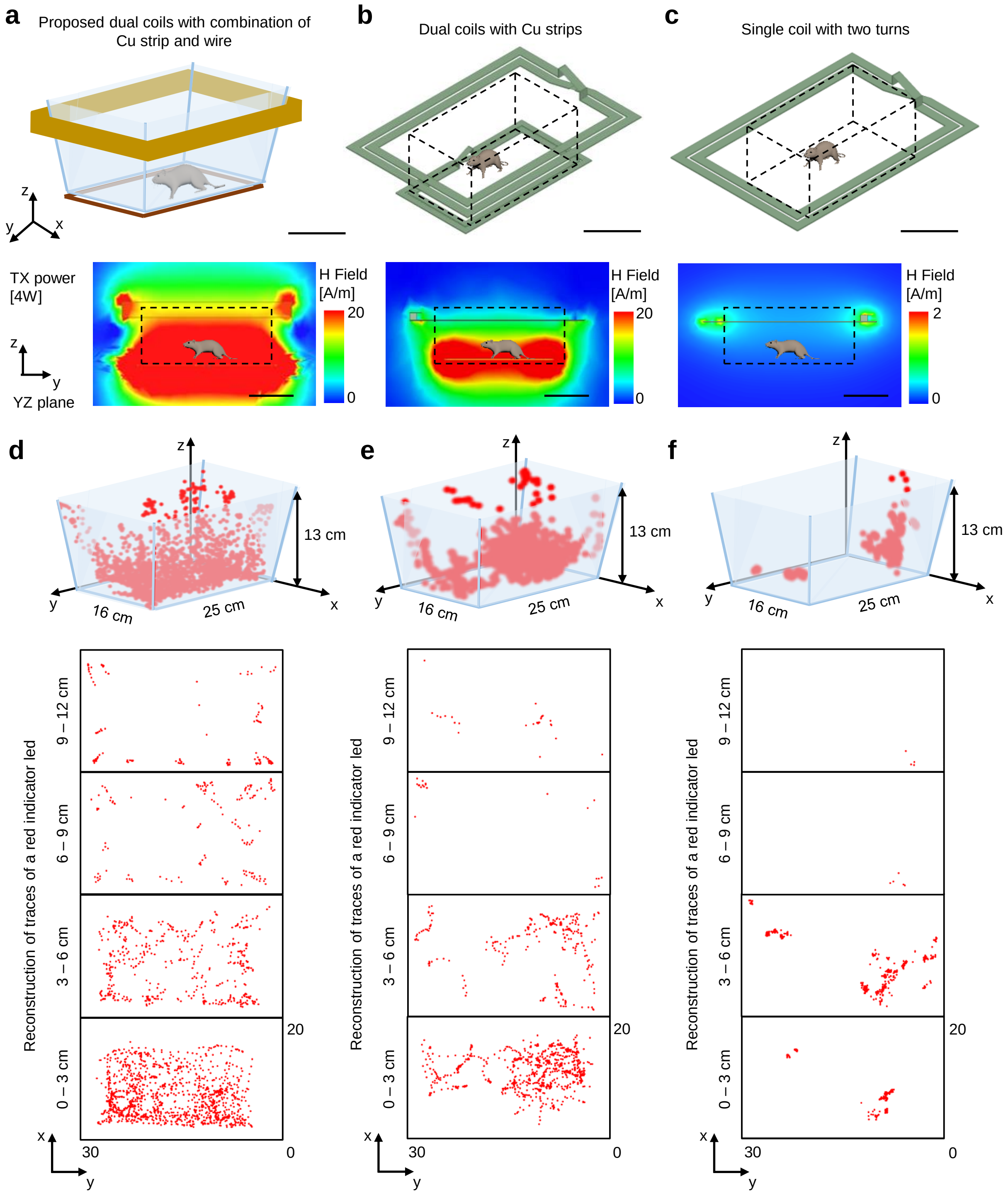

Supplementary Figure 10. Illustration of an antenna layout (top) and distributions of magnetic field in a cage (bottom) for the proposed (**a**), dual coil with Cu strips (**b**), and single coil antenna structure (**c**), respectively; scale bar 10 cm. Reconstruction of traces of an operating indicator led (top) and cross-sectional view of it at each ranges of height for the proposed (**d**), dual coil with Cu strips (**e**), and single coil antenna structure (**f**), respectively.

Supplementary Figure 11.

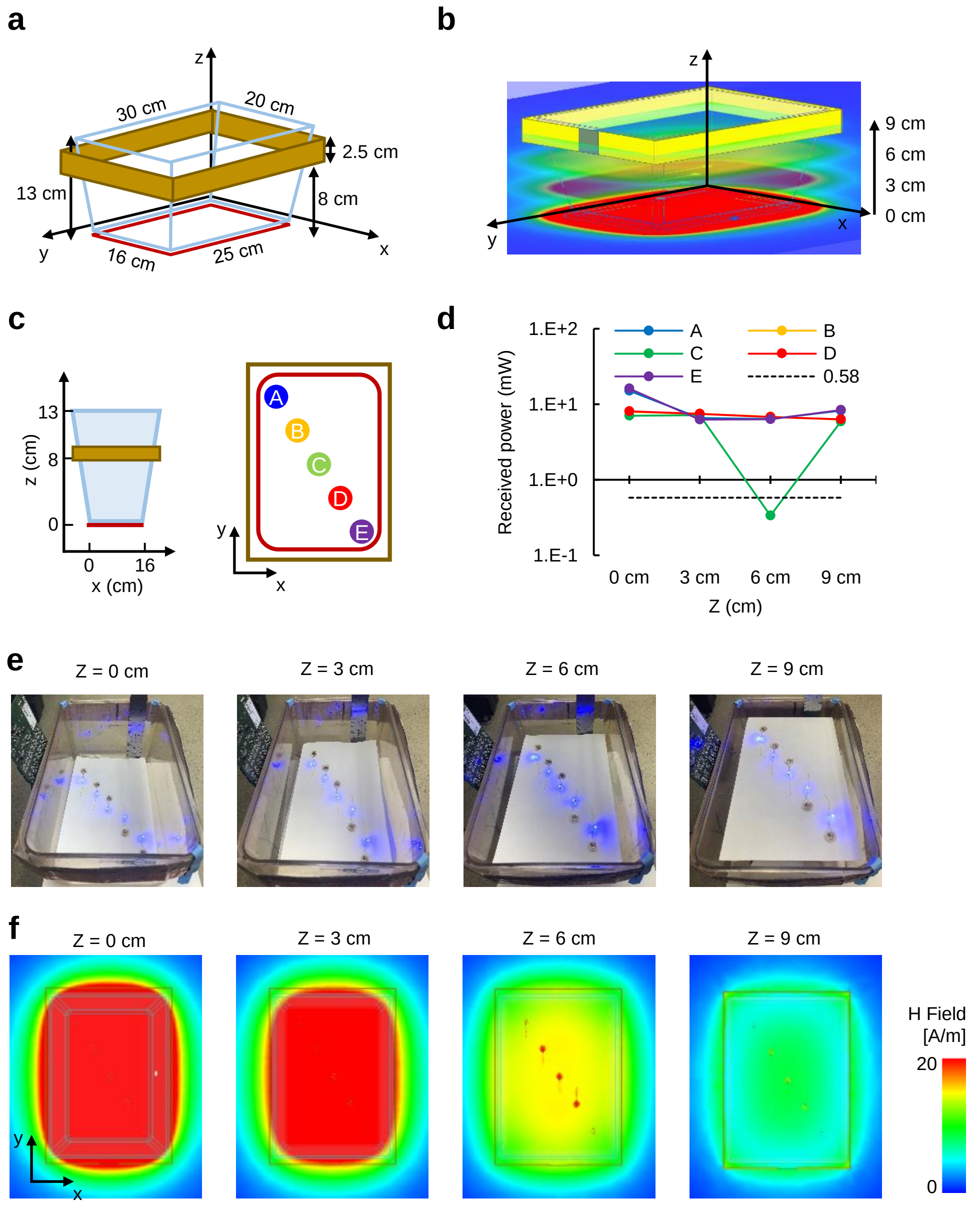

Supplementary Figure 11. (**a**) Representative image of a cage with the proposed antenna coils installed. (**b**) Distributions of electromagnetic field in a cage at each distances from the bottom. (**c**) Illustration of measurement setup. (**d**) Measurements of output power at 5 different locations of the cage as a function of the distance along the z axis. Dotted lines indicate the threshold electrical power (0.58 mW) required for the activation of light-sensitive opsins; 0.58 mW electrical power corresponds to an optical power of 10.12 mW/mm^2. (**e**) Representative images of wireless operation in the cage at each distance. (**f**) Corresponding distributions of electromagnetic field in the cage at each distance from the bottom.

Supplementary Figure 12.

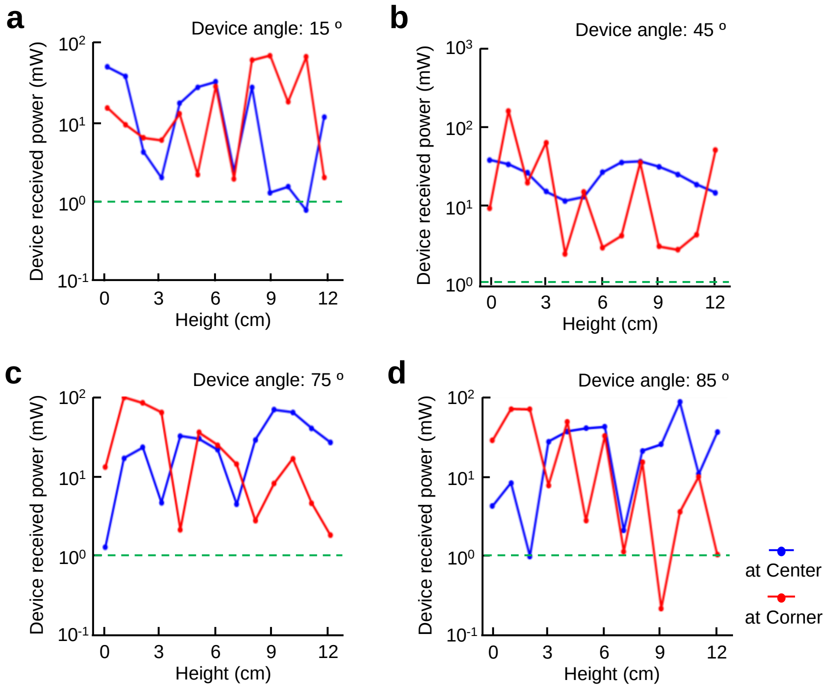

Supplementary Figure 12. Plots of a residual dependence of transmitted power on relative orientation angle between the transmission antenna and the implantable device as a function of the angle, 15 º (**a**), 45 º (**b**), 75 º (**c**), and 85 º (**d**). Green dotted lines indicate threshold electrical power level of 1 mW required for activation of light-sensitive proteins; 1mW electrical power corresponding to an optical power of 17.44 mW/mm^2.

Supplementary Figure 13.

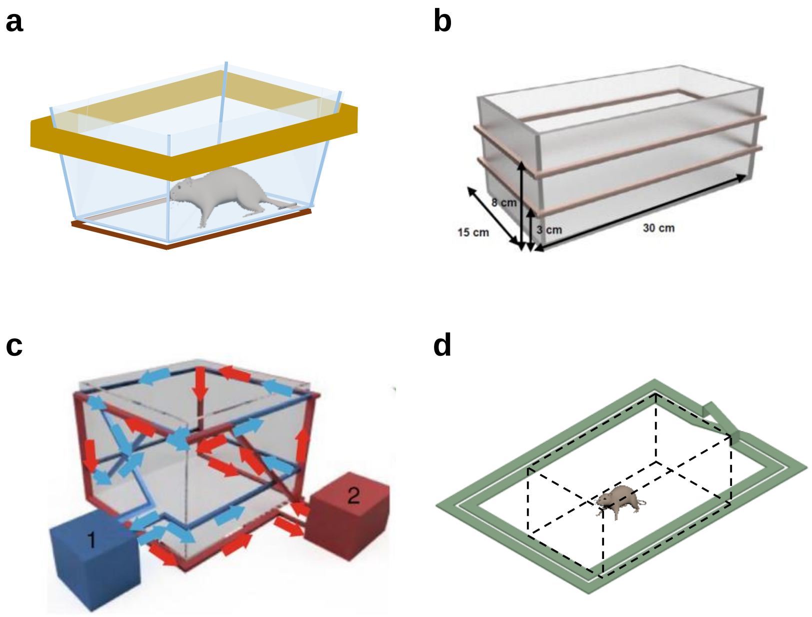

Supplementary Figure 13. Comparison of wireless coverage for the proposed antenna coil and other antenna coil systems. Representative image of the proposed antenna coil (**a**), single coil & dual layered with Cu wire (**b**), a tilted antenna design with a time division multiplexing scheme (**c**), dual coil antenna with Cu strips, and single coil with two turns (**d**).

Supplementary Figure 14.

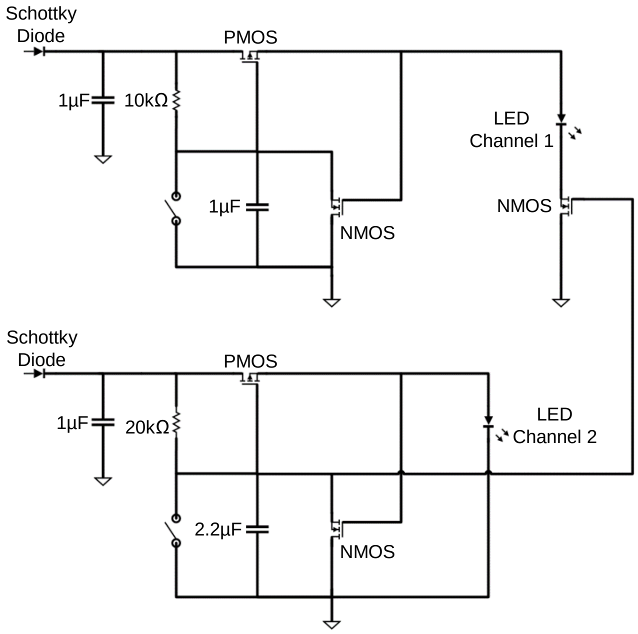

Supplementary Figure 14. Circuit diagram of the proposed, scalable multimodal wireless gastric optogenetic implant for multiple organ control.

Supplementary Figure 15.

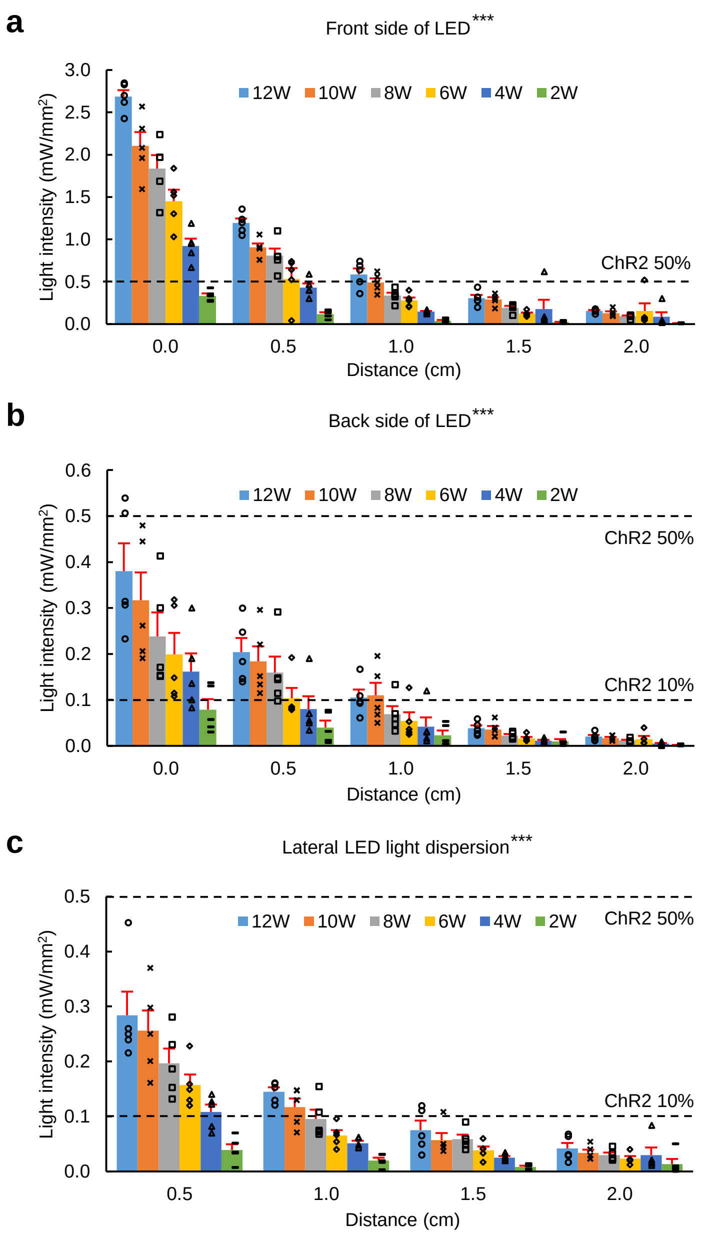

Supplementary Figure 15. Light intensity measurements during varying RF wireless powering ($p < 0.001$) of the gastric optogenetic device ($n = 5$) and varying distances ($p < 0.001$) from the LED. Measurements were taken from the front side (**a**), back side (**b**), and lateral side of the LED (**c**). Bar graphs are mean ± SEM. Statistical comparisons were made two-way repeated-measures ANOVA; *** $p < 0.001$.

Supplementary Figure 16.

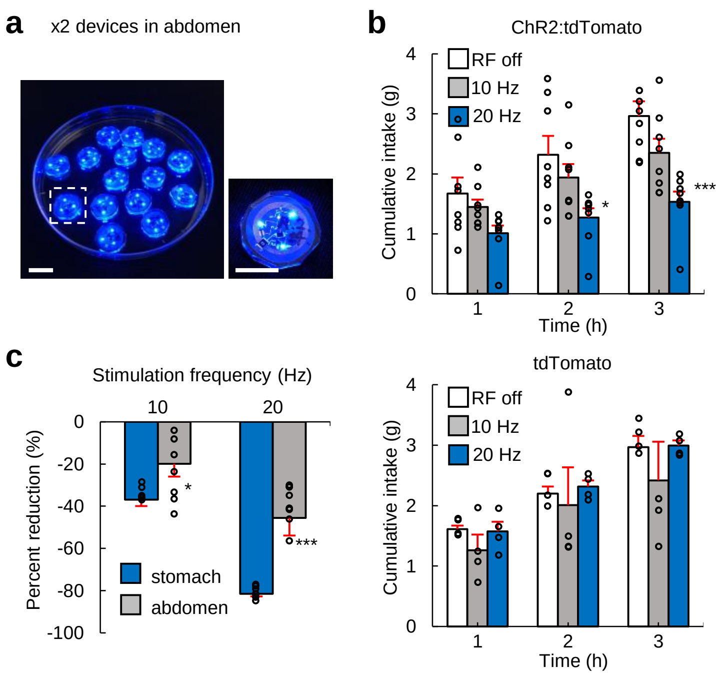

Supplementary Figure 16. Abdominal activation of *Calca*+ vagal afferent fibers. (**a**) Picture showing wirelessly powered LED device; two of these devices were inserted into the abdomen of *Calca*-Cre transgenic mice with left nodose ganglion injection of AAV9-DIO-ChR2:tdTomato or AAV9-DIO-tdTomato control virus; scale bar 5 mm. (**b**) top, frequency dependent suppression of food intake during ChR2 activation of vagal afferents (n = 8), bottom, no appetite suppression in tdTomato control group (n = 4) (p = 0.80). (**c**) Percent reduction of food intake (compared to RF off) during 10 and 20 Hz stimulation of *Calca*+ vagal afferent endings using the stomach LED implant or non attached LEDs (ChR2, n = 8 per group) (interaction, p = 0.01). Bar graphs are mean ± SEM. Statistical comparisons were made two-way repeated-measures ANOVA, Tukey's post hoc; * p < 0.05; ** p < 0.01; *** p < 0.001.

Supplementary Figure 17.

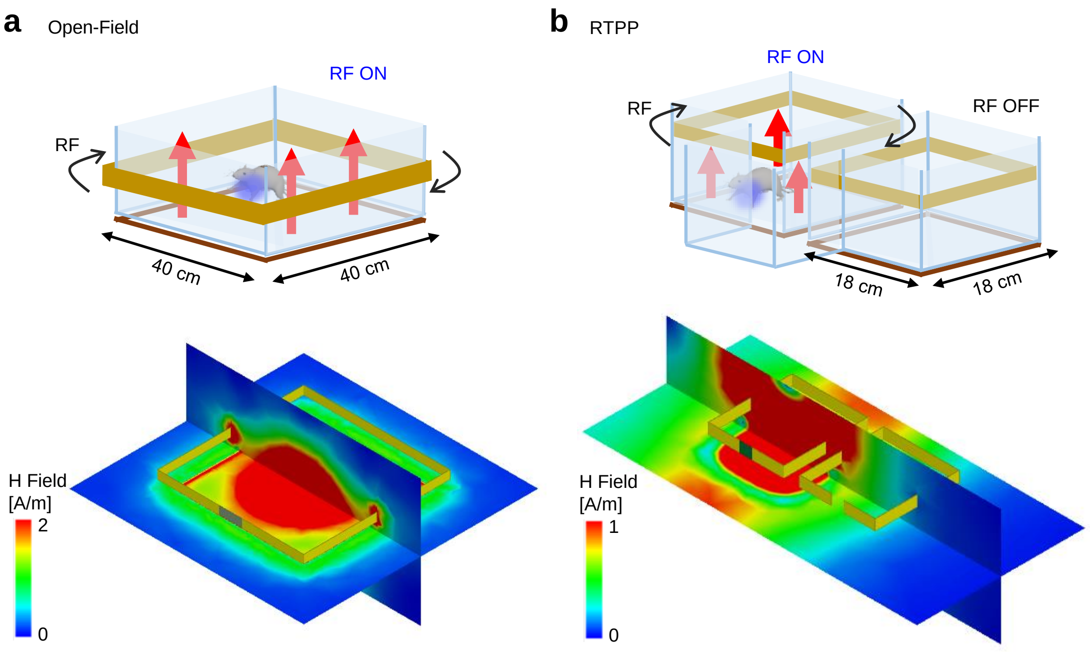

Supplementary Figure 17. Schematic illustration of an experimental assay (top) and distributions of electromagnetic field in the assay (bottom) for open-field (**a**) and RTPP (**b**), respectively.

Supplementary Figure 18.

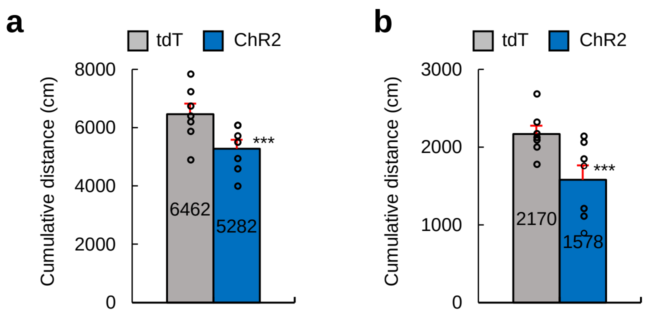

Supplementary Figure 18. Locomotor activity comparison in the assays for RTPP (**a**), and open-field (**b**), respectively. Both conducted for 30 minutes and n = 7 per each group. Bar graphs are mean ± SEM. Statistical comparison was made using two-tailed t-test; *** $p < 0.001$.

Supplementary Table 1

Supplementary Table 1. Summary of procedures for fabrications

Process		Purpose	Required time for 10 devices	Equipment	Progress level (%)
**1**	Preparation of photoresist coated glass	Sampling for transfer	1 hours	Clean room Spin-coater	10
**2**	Baking	Stabilization, remove solvent	0.5 hours	Clean room Hotplate	20
**3**	Photo lithography	Patterning for stretchable circuits	1 hours	Clean room Mask aligner	40
**4**	Photoresist development		0.5 hours	Clean room	50
**5**	Copper etching		1 hours	Clean room	60
**6**	Baking	Stabilization, remove solvent	0.5 hours	Clean room Hotplate	65
**7**	Components transfer	Active components integration	5 hours	Soldering Iron Microscope	80
**8**	PDMS encapsulation	System packaging	10 hours	Vacuum oven	100

Supplementary Table 2

Supplementary Table 2. Summary of customized TX antenna specification

Cage image		Cage dimension*	Distance of top & bottom coils	Antenna dimension*		Inductance of Antenna		Required capacitance	
				Top**	Bottom***	Top**	Bottom***	Top**	Bottom***
**1**	Home cage 1 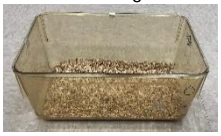	20 x 30 x 13	8 cm	19 x 29	16 x 25	660 nH	670 nH	120 pF	167 pF
**2**	Home cage 2 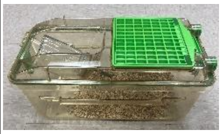	20 x 38 x 16	12 cm	20 x 37	16 x 29	780 nH	780 nH	102 pF	147 pF
**3**	Home cage 3 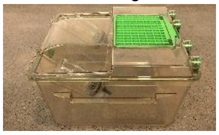	34x 39 x 21	6 cm	34 x 36	29 x 29	940 nH	1.00 µH	85 pF	110 pF
**4**	RTPP 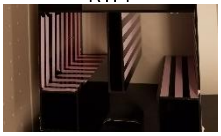	Inner box<br>18 x 18 x 30<br><br>Outer box<br>41 x 41 x 30	14 cm	18 x 18	16 x 16	485 nH	580 nH	164 pF	189 pF
**5**	Open field 1 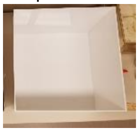	40 x 40 x 30	3 cm	41 x 41	37 x 37	1.04 µH	1.42 µH	74 pF	79 pF
**6**	Open field 2 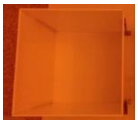	28 x 28 x 30	5 cm	30 x 30	26 x 26	760 nH	1.23 µH	100 pF	92 pF

All values are $\pm$5% tolerance.
*****dimension: (width) x (length) x (height) or (width) x (length); unit: cm
******Top coil material: Copper stripes (2.54 cm wide; 0.635 mm thick)
*******Bottom coil material: 8-Gauge bare copper wire
