# Supplementary material for: Organ-specific, multimodal, wireless optoelectronics for high-throughput phenotyping of peripheral neural pathways
Source: Nat Commun. 2021 Jan 8;12:157. doi: 10.1038/s41467-020-20421-8 (PMC7794361; doi:10.1038/s41467-020-20421-8)
Supplement: Supplementary file 6 — Reporting Summary [file 41467_2020_20421_MOESM6_ESM.pdf]

nature research

Corresponding author(s): Sung Il Park
Last updated by author(s): Nov 5, 2020

Reporting Summary

Nature Research wishes to improve the reproducibility of the work that we publish. This form provides structure for consistency and transparency in reporting. For further information on Nature Research policies, see our Editorial Policies and the Editorial Policy Checklist.

Statistics

For all statistical analyses, confirm that the following items are present in the figure legend, table legend, main text, or Methods section.

n/a	Confirmed	
☐	☒	The exact sample size (*n*) for each experimental group/condition, given as a discrete number and unit of measurement
☐	☒	A statement on whether measurements were taken from distinct samples or whether the same sample was measured repeatedly
☐	☒	The statistical test(s) used AND whether they are one- or two-sided<br>*Only common tests should be described solely by name; describe more complex techniques in the Methods section.*
☒	☐	A description of all covariates tested
☐	☒	A description of any assumptions or corrections, such as tests of normality and adjustment for multiple comparisons
☐	☒	A full description of the statistical parameters including central tendency (e.g. means) or other basic estimates (e.g. regression coefficient) AND variation (e.g. standard deviation) or associated estimates of uncertainty (e.g. confidence intervals)
☐	☒	For null hypothesis testing, the test statistic (e.g. *F*, *t*, *r*) with confidence intervals, effect sizes, degrees of freedom and *P* value noted<br>*Give P values as exact values whenever suitable.*
☒	☐	For Bayesian analysis, information on the choice of priors and Markov chain Monte Carlo settings
☒	☐	For hierarchical and complex designs, identification of the appropriate level for tests and full reporting of outcomes
☒	☐	Estimates of effect sizes (e.g. Cohen's *d*, Pearson's *r*), indicating how they were calculated

Our web collection on statistics for biologists contains articles on many of the points above.

Software and code

Policy information about availability of computer code

Data collection	For thermal assessment of devices, we used software IRBIS3 package.
Data analysis	For numerical electromagentic simulations, we used a finite element-method analysis tool (Ansys Electromagnetics Suite 17-HFSS, Ansys).<br>We used a commercial finite element-method analysis tool (Abaqus/CAE 2018, Dassault Systems) for the 3-D modeling of the mechanics.<br>Custom C code was used to program on the multiplexer controller (nRF52832 Development Kit, Nordic semiconductor). This code was based on C code libraries from Nordic (nRF5_SDK_13.0.0_04a0bfd) and Keil uVision 5 IDE(uVision V5.23.0.0).<br>The 3-D continuous traces image reconstructed by using custom scripts in Python (version 3.7.3-64bit, Spyder 3.3.6 IDE). This customized codes are available at https://github.com/parkgroup-tamu/3d_reconstruction.<br>The food-monitoring software (BioDAQ, v.2.2) were used for meal-pattern analysis, and feeding records were analyzed using BioDAQ Viewer (software v.2.2.01).<br>The video-tracking software (EthoVision XT 10, Noldus) was used for generating traces of RTTP and Open-field assay.<br>Statistics data were analyzed using Prism 5.0 (GraphPad Software).

For manuscripts utilizing custom algorithms or software that are central to the research but not yet described in published literature, software must be made available to editors and reviewers. We strongly encourage code deposition in a community repository (e.g. GitHub). See the Nature Research guidelines for submitting code & software for further information.

Data

Policy information about availability of data

All manuscripts must include a data availability statement. This statement should provide the following information, where applicable:

- Accession codes, unique identifiers, or web links for publicly available datasets
- A list of figures that have associated raw data
- A description of any restrictions on data availability

The main data supporting the results in this study are available within this article and its supplementary information. The source data underlying Fig. 1e-g, 3a, b, 4b-c, e, h, j and Supplementary Figs. 4a-c, 5, 6a, b, d, e, 11d, 12a-d, 15a-c, 16b, c, 18a, b are provided as a Source Data file.

Field-specific reporting

Please select the one below that is the best fit for your research. If you are not sure, read the appropriate sections before making your selection.

☐ Life sciences ☒ Behavioural & social sciences ☐ Ecological, evolutionary & environmental sciences

For a reference copy of the document with all sections, see nature.com/documents/nr-reporting-summary-flat.pdf

Behavioural & social sciences study design

All studies must disclose on these points even when the disclosure is negative.

Study description	Quantification of stomach LED device durability and quantification of feeding behavior during stomach vagal afferent stimulation with optogenetics.
Research sample	CalcaCre:GFP mice (C57Bl/6 background) were generated and maintained as described in Carter et al. 2013. Following surgery, mice were singly housed with ad libitum access to standard chow diet (LabDiet 5053) in temperature- and humidity-controlled facilities with 12-h light/dark cycles. Both male and female mice were used for behavioral experiments to provide representative data of both sexes.
Sampling strategy	Groups sizes were determined using power analysis and expected variability based on past food intake behavior experiments.
Data collection	Experimenter was blind to treatment during food intake measurements. Data was collected by measuring difference in food weight using a scale and tabulating measurements using pen and paper.<br>Experimenter was blind to treatment during device testing. Data was collected by measuring optical intensity using a photometer and temperature changes with IR camera using pen and paper.
Timing	Mice were housed in 12-12 light dark cycle. Food was removed an hour before lights of (ZT 23 h) and refed the following morning around ZT 14 h (two hours after lights on). Other behavioral tests were conducted between ZT 14-18 h. Behavioral experiments were conducted between February-May and October-December 2019.
Data exclusions	Behavior data was excluded if there was no expression of fluorescent reporter, suggesting missed virus injection, or non-operational LED device by the end of all experiments. Nearly all data was included. Two mice injected with AAV9-DIO-ChR2:tdTomato were excluded from behavioral analysis due to little (2-3 neurons infected per section) or no virus infection as determined by visualizing the fluorescent reporter, tdTomato.
Non-participation	The item is not relevent to our study.
Randomization	Experimental groups counterbalanced, as much as possible, based on littermate, sex, and weight.<br>No participants

Reporting for specific materials, systems and methods

We require information from authors about some types of materials, experimental systems and methods used in many studies. Here, indicate whether each material, system or method listed is relevant to your study. If you are not sure if a list item applies to your research, read the appropriate section before selecting a response.

Materials & experimental systems

n/a	Involved in the study	
☒	☐	Antibodies
☒	☐	Eukaryotic cell lines
☒	☐	Palaeontology and archaeology
☐	☒	Animals and other organisms
☒	☐	Human research participants
☒	☐	Clinical data
☒	☐	Dual use research of concern

Methods

n/a	Involved in the study	
☒	☐	ChIP-seq
☒	☐	Flow cytometry
☒	☐	MRI-based neuroimaging

Animals and other organisms

Policy information about studies involving animals; ARRIVE guidelines recommended for reporting animal research

Laboratory animals	Male and female C57/Bl6 mice, age 2-4 months were used.
Wild animals	The study did not involve wild animals.
Field-collected samples	The study did not involve samples collected from the field.
Ethics oversight	All animal care and experimental procedures were approved by the Institutional Animal Care and Use Committee at the University of Washington.

Note that full information on the approval of the study protocol must also be provided in the manuscript.
