# Supplementary material for: Organ-specific, multimodal, wireless optoelectronics for high-throughput phenotyping of peripheral neural pathways
Source: Nat Commun. 2021 Jan 8;12:157. doi: 10.1038/s41467-020-20421-8 (PMC7794361; doi:10.1038/s41467-020-20421-8)
Supplement: Supplementary file 7 — Description of Additional Supplementary Files [file 41467_2020_20421_MOESM7_ESM.pdf]

Title: Supplementary Movie 1.
Description: *In vitro* demonstration of low power 8-channel wireless power TX system.

Title: Supplementary Movie 2.
Description: *In vivo* demonstration of low power 8-channel wireless power TX system.

Title: Supplementary Movie 3.
Description: *In vivo* demonstration of dual-channel function: A single pulse from the TX system switches from channel 1 (Green) to channel 2(Blue).

Title: Supplementary Movie 4.
Description: *In vivo* demonstration of optogenetic control of food intake: appetite suppression group (Left) and the control group without ChR2 (Right).
